# Metabolic Factors Related to Interpersonal Dysfunction in Acromegaly: A Nationwide Cross‐Sectional Study in China

**DOI:** 10.1111/cns.70607

**Published:** 2025-09-15

**Authors:** Shashi Kiran Tagilapalli, Zongming Wang, Yuechu Lucinda Lu, Guofeng Zhang, Weijie Su, Zhentian Wu, Jiaming Wang, Qing Rao, Haijun Wang, Dongsheng He, Yonggao Mou, Shun Yao, Yanmei Tie, Wenli Chen

**Affiliations:** ^1^ Center for Pituitary Tumor Surgery, Department of Neurosurgery, the First Affiliated Hospital Sun Yat‐Sen University Guangzhou Guangdong China; ^2^ Department of Neurosurgery, the Sixth Affiliated Hospital Sun Yat‐Sen University Guangzhou Guangdong China; ^3^ Biomedical Innovation Center, the Sixth Affiliated Hospital Sun Yat‐Sen University Guangzhou Guangdong China; ^4^ Morrissey College of Arts and Sciences Boston College Boston Massachusetts USA; ^5^ Department of Statistics, the First Affiliated Hospital Sun Yat‐Sen University Guangzhou China; ^6^ Zhongshan School of Medicine Sun Yat‐Sen University Guangzhou Guangdong China; ^7^ Department of Neurosurgery Sun Yat‐Sen University Cancer Center Guangzhou China; ^8^ Department of Neurosurgery Brigham and Women's Hospital, Harvard Medical School Boston Massachusetts USA

**Keywords:** acromegaly, cardiovascular dysfunction, high growth hormone levels, interpersonal problems, quality of life

## Abstract

**Objective:**

To identify the clinical manifestations, metabolic factors, and comorbidities independently associated with interpersonal dysfunction in Chinese acromegaly patients.

**Methods:**

We analyzed clinical, cognitive, and comorbidity data from 585 acromegaly patients across 112 tertiary hospitals in China (July 1995–December 2018). Interpersonal difficulties were quantified using the Inventory of Interpersonal Problems‐Distress (IIP‐D) and dichotomized into low (< 17) and high (≥ 17) groups. Group differences were tested with nonparametric tests. Supervised machine‐learning models were developed to predict features associated with the high IIP‐D group, with performance evaluated via five‐fold cross‐validation. The top‐performing model was further validated on the held‐out data set and the feature importance analysis identified the key predictors. Exploratory hierarchical clustering (Ward's method) was used to explore symptom groupings, though sampling adequacy was limited.

**Results:**

Patients with high interpersonal distress exhibited significantly higher preoperative growth hormone (GH), frontal bossing, palpitations, and cognitive impairment (all *p* < 0.05). Among machine‐learning models, extreme gradient boosting (XGBoost) demonstrated the highest performance, area under the curve (AUC = 0.868 in across‐validation), and maintained strong accuracy in final testing (AUC = 0.873). Key independent predictors included frontal bossing, palpitations, cardiomyopathy, disease duration, preoperative GH, acral enlargement, arrhythmia, and atrial fibrillation.

**Conclusion:**

Physical disfigurement, palpitations, cardiac comorbidities, and elevated GH levels independently predict high IIP‐D in acromegaly. Integrating systematic psychosocial screening into neuroendocrine care, alongside referral to psychoneuroendocrine teams, may help mitigate social disability and improve quality of life.

## Graphical abstract

Metabolic and physical factors—specifically frontal bossing, cardiovascular comorbidities, and elevated preoperative GH levels—are independent predictors of severe interpersonal dysfunction in Chinese patients with acromegaly. These findings underscore the need for integrating systematic psychosocial screening into routine neuroendocrine management to optimize patient outcomes.

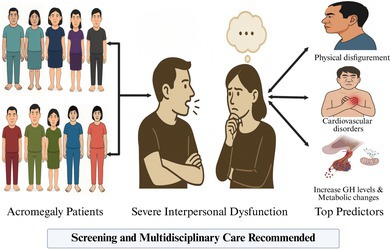

## 1 Introduction

Acromegaly is a rare chronic endocrinopathy caused by sustained hypersecretion of growth hormone (GH) and consequent insulin‐like growth factor 1 (IGF‐1) excess. Prolonged exposure to these hormones leads to progressive somatic disfigurement, widespread metabolic dysregulation, and a high burden of physical and cognitive symptoms [1, 2, 3]. Beyond its well‐recognized peripheral manifestations, GH excess disrupts cerebral vasculature and perfusion, promoting endothelial dysfunction through oxidative stress and aberrant endothelial proliferation, thereby amplifying the risk of cardiovascular [4, 5, 6], cerebrovascular, and respiratory diseases [5, 7]. Delayed diagnosis or suboptimal control is often associated with a higher risk of hypertension, cardiomyopathy, stroke, and Obstructive Sleep Apnea (OSA), collectively shortening life expectancy [8, 9, 10, 11, 12].

Even after modern medical, surgical, and radiotherapeutic advances, post‐achieved biochemical control, many patients still report persistent impairments in health‐related quality of life (HRQoL) [13, 14, 15, 16, 17]. Chronic joint pain, fatigue, cardiopulmonary complications, and mild‐to‐moderate cognitive deficits are common symptoms [17, 18, 19]. Xie et al. reported cerebral microbleeds (CMBs) in ~29% of acromegalic patients versus ~5% of controls, and OSA independently predicted CMBs presence, indicating disordered cerebral perfusion in the cognitive decline observed in acromegaly [15, 20]. These neurological sequelae have tangible psychosocial consequences.

Among these psychological outcomes, interpersonal dysfunction remains critical yet underexplored. Difficulties in self‐functioning and social relationships can erode emotional resilience, hinder community reintegration, and jeopardize adherence to long‐term treatment [3, 21, 22, 23]. These issues are comprehensively captured by the Inventory of Interpersonal Problems–Difficulty (IIP‐D) scale robustly, a tool that has shown clinical relevance in the context of chronic disease. Prior work by Zimmermann et al. identified an association between elevated IIP‐D scores and coronary heart disease and malignancy history, emphasizing the complex interplay between physical morbidity and interpersonal impairments [21]. However, the relative contributions of distinct physical manifestations (e.g., disfiguring skeletal changes and cardiovascular symptoms), metabolic dysfunction, and cognitive impairments to interpersonal dysfunction in acromegalic conditions have not been systematically examined.

To bridge this knowledge gap, we conducted a nationwide cross‐sectional study using data from the China Acromegaly Patient Association (CAPA) registry [24]. We aimed to identify the metabolic factors, clinical manifestations, cognitive deficits, and comorbidities that were most strongly associated with interpersonal difficulties. Clarifying these predictors will inform early psychoneurological assessment and guide multidisciplinary care to enhance social functioning and overall QoL in this vulnerable population.

## 2 Methods

### 2.1 Study Design and Population

**FIGURE 1:**
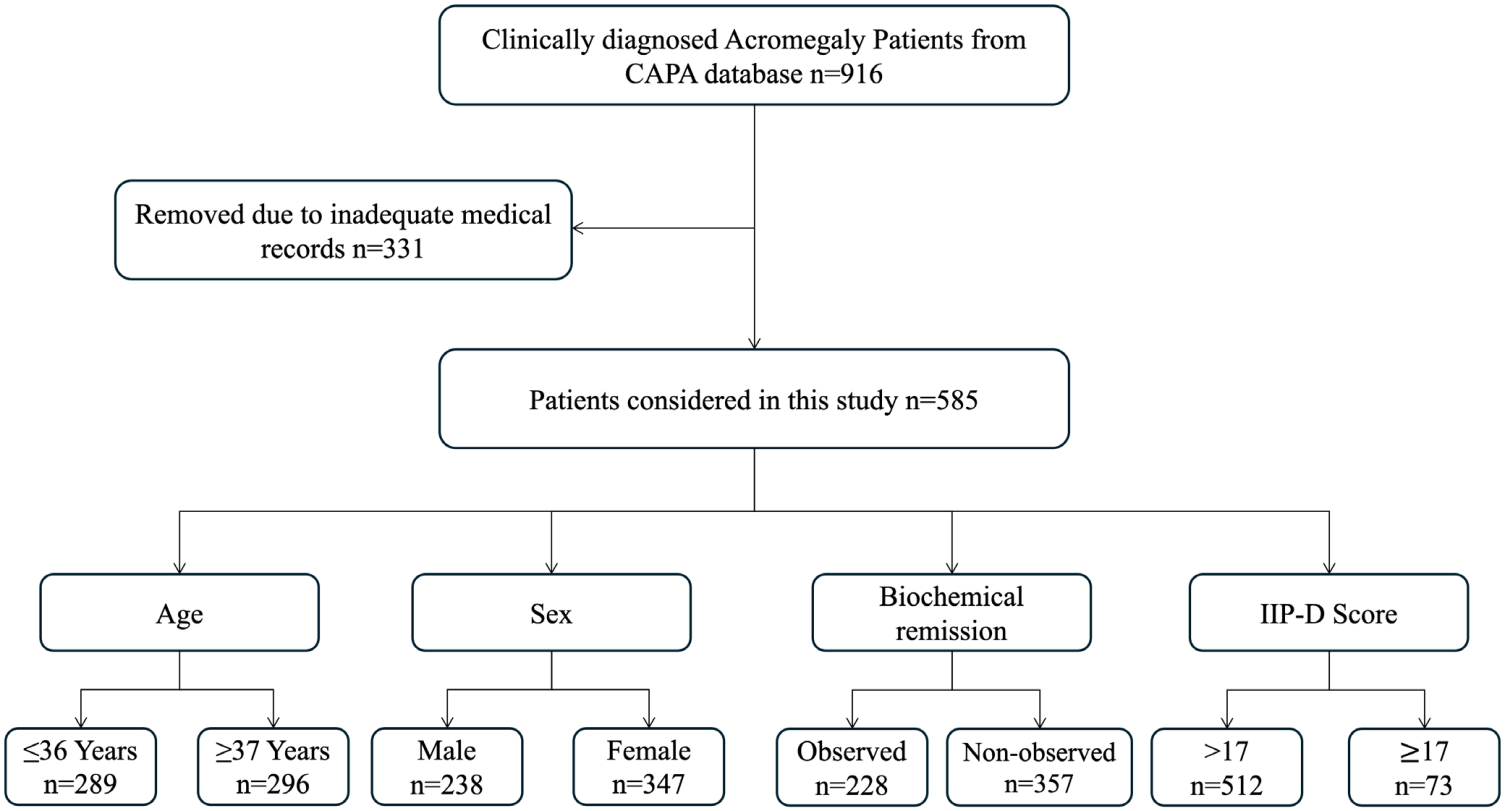
Flowchart of patient selection and subgroup classification. Of 916 acromegaly patients, 585 were included in the final analysis based on data completeness. Patients were stratified by age, sex, biochemical remission, and IIP‐D score groups.

We performed a nationwide, cross‐sectional, retrospective analysis of the CAPA registry, which captures standardized clinical information from 112 tertiary hospitals across China, including demographic variables (age, sex, and geographic region), clinical manifestations, hormonal and biochemical parameters (standardized to WHO IS 98/574), radiological findings, pathology reports, surgical notes, and longitudinal follow‐up information. All patients diagnosed with acromegaly between June 1995 and December 2018 were screened (*n* = 916). Eligible participants had histologically confirmed disease, a history of transsphenoidal surgery, and possessed complete peri‐operative and biochemical follow‐up data. Patients who had not undergone surgery or whose records were incomplete were excluded, yielding a final cohort of 585 patients (Figure 1). The patients' characteristics are summarized in (Table S1). To ensure data quality, all site investigators completed a mandatory web‐based training module on the CAPA platform before data entry.

### 2.2 Inventory of Interpersonal Problems—Difficulty (IIP‐D)

The IIP‐D questionnaire serves as a validated self‐report assessment tool that helps identify and quantify interpersonal difficulties. This investigation utilized the shortened German version of the IIP‐D, which contains 32 items based on a circumplex model to assess control/dominance and affection dimensions, and has been widely used in research [21, 22, 23, 25, 26, 27], and offers the advantage of comprehensive coverage of interpersonal domains in a shorter form. The model divides interpersonal difficulties into eight specific subscales which include domineering; vindictive/competitive; cold/denying; introverted/socially avoidant; non‐assertive/insecure; exploitable/permissive; nurturant (encompassing behaviors indicative of both physical and emotional caregiving capacities); and intrusive (characterized as disruptive and unwelcome interpersonal behaviors). The respondents rated each item using a Likert‐type scale ranging from 0 (“not at all”) to 4 (“extremely”). The scores for each subscale were obtained by calculating the average of all items within their respective domains. Standardized scores were used to compare the results with the normative German population, which included participants aged 14–98 years. The IIP‐D composite score for each participant was determined by calculating the mean score from all eight interpersonal subscales (Equation 1).

(1) $$\mathrm{IIP}-D\;\text{Composite Score}=\frac{1}{8}\sum_{i=1}^{8}\mathrm{Si}$$

This questionnaire was adapted for use in the Chinese population through a standardized forward‐backward translation procedure conducted by clinical practitioners and bilingual experts (D.H., Y.T., and S.Y.) to ensure cultural and linguistic equivalence.

### 2.3 Definitions

#### 2.3.1 Biochemical Status

Postoperative biochemical remission was defined based on consensus biochemical criteria. Acromegaly was deemed active if any of the following applied: (i) random serum GH level ≥ 2.5 μg/L or nadir GH level ≥ 1 μg/L during a 75 g oral glucose tolerance test (OGTT); or (ii) standardized IGF‐1 level above the age‐ and sex‐adjusted upper limit of normal derived from 2791 healthy Chinese adults (1339 men, 1452 women) [10, 24, 28].

#### 2.3.2 Interpersonal Dysfunction

The Inventory of Interpersonal Problems (IIP‐D) comprises eight subscales, with a total composite score of 32. In this study, patients were stratified based on the median IIP‐D score (median = 16). A score of ≥ 17, reflecting values above the median, was defined as the high IIP‐D group, indicative of clinically relevant interpersonal dysfunction, and was used as the threshold for subsequent analyses.

### 2.4 Statistical Analysis

Continuous variables are presented as mean ± standard deviation (SD) or median (interquartile range, IQR), and categorical variables as counts (percentages). Between‐group differences for continuous variables were evaluated using the Mann–Whitney *U* test, while categorical variables were analyzed via the Chi‐squared test or Fisher's exact test.

Before exploratory factor analysis, the sample's adequacy and the data's suitability were assessed using the Kaiser–Meyer–Olkin (KMO) measure and Bartlett's test of sphericity. All statistical analyses were performed using Python (version 3.12.1; Python Software Foundation, https://www.python.org/) with relevant libraries including NumPy (version 2.0), pandas (version 3.0), SciPy (version 1.16), and scikit‐learn (version 1.7) and R v4.4.3 (R Foundation for Statistical Computing, Vienna, Austria). The two‐sided *P* values < 0.05 were considered statistically significant.

### 2.5 Feature Selection and Classification Model

Supervised machine‐learning models were developed using clinical and demographic features associated with the high IIP‐D (≥ 17). To reduce dimensionality and identify the most discriminatively associated features, principal component analysis (PCA) was performed. Model training/validation for Logistic Regression, Random Forest, Gradient Boosting, extreme gradient boosting (XGBoost), and multilayer perceptron (MPL) models with a stratified 5‐fold cross‐validation strategy was employed to estimate model generalizability and performance metrics (AUC, accuracy, sensitivity, specificity, macro‐F1). Discrimination was quantified with receiver operating characteristic (ROC) curves and the area under the ROC curve (AUC). The best‐performing model, identified during cross‐validation, was selected for final testing.

Hierarchical agglomerative clustering (Ward's linkage, Euclidean distance) was applied to identify clinically significant subgroups or symptom clusters related to interpersonal dysfunction. Associations among quantitative variables were examined using Spearman's and Pearson's correlations, and the results were visualized accordingly. The weak, moderate, and strong monotonic relationships are defined by the magnitude of |*p*|: < 0.39, 0.40 to 0.59, and > 0.60.

## 3 Results

### 3.1 Baseline Patient Characteristics

Among the 585 eligible patients, 347 were women (59.5%). Age at diagnosis ranged from 31 to 44 years, with women presenting at older ages than men. The most common presenting features were coarsening of facial features (93.6%), acral enlargement (92.8%), headache (57.6%), hyperhidrosis (56.7%), frontal bossing (49.4%), and visual impairment (43.4%). Headaches were more frequently reported among patients with macroadenomas than micro or giant adenomas (56.9%), but the difference was insignificant.

Cognitive impairment, predominantly memory deficits, was observed in 76% of patients. Osteoarticular disease was the leading comorbidity (57.1%), followed by sexual dysfunction (38.2%) and OSA (24.2%). Among women, menstrual irregularity was notably common (66.3%).

In the radiological features, macroadenomas predominated (67.8%), whereas microadenomas and giant adenomas accounted for 22.2% and 9.9%, respectively. There was no statistically significant difference in tumor size between genders. Pituitary apoplexy was documented in 9.6% of patients, slightly more often in women (10.1% vs. 8.9%). Cavernous sinus invasion was the most frequent invasive pattern (63.5%), followed by encasement of the internal carotid artery (13.7%) and extension into the sphenoid sinus (10.6%) (Table S1).

### 3.2 Biochemical Condition and Interpersonal Difficulty

Of 585 patients in the study, 228 (38.9%) achieved biochemical remission, and 357 (61.1%) were biochemically active. According to the results of Fisher's test (*χ* ^2^ = 0.00016, *p* = 0.990), demonstrating that no significant statistical relationship was found between biochemical remission and interpersonal dysfunction. The total number of high IIP‐D group patients was 73; of these, 44 (60.3%) patients are biochemically active, and 29 (39.7%) patients achieved initial biochemical remission.

### 3.3 KMO and Bartlett's Test

Prior to exploratory factor analysis, the adequacy of the sample and the suitability of the data were assessed using the KMO measure and Bartlett's test of sphericity. The KMO value obtained was 0.438. Nevertheless, Bartlett's test of sphericity was statistically significant (*χ* ^2^ = 18,324.37; df = 1653; *p* < 0.001), confirming that the correlations among the variables were sufficient to justify the exploratory analysis.

### 3.4 Clinical Correlations of Interpersonal Difficulty

Coarsening of facial features, zygomatic hypertrophy, headaches, frontal bossing, and visual abnormalities were each associated with higher IIP‐D scores (all *p* < 0.001). Cognitive impairments, including learning disabilities, hallucinations, memory decline, and impaired spatial perception, were likewise related to elevated IIP‐D scores (*p* = 0.006). Palpitations, colorectal disorders, menstrual irregularities, sexual dysfunction, and obesity showed similar associations (Table 1).

**TABLE 1:** Comparison of symptom‐related quality of life scores between patients with and without clinical or cognitive dysfunction.

	Symptoms absence group (*n*)	Symptoms presence group (*n*)	*P*
	M (P_25_, P_75_)	M (P_25_, P_75_)	
Coarsening of facial features	37	548	0.396
	7.0 (2.5, 11.0)	8 (3.0, 13.0)	
Acral enlargement	42	543	< 0.001**
	4 (0.0, 8.0)	8 (3.0, 14.0)	
Zygomatic bone hypertrophy	219	366	< 0.001**
	6 (2.0, 11.0)	8 (3.7, 14.0)	
Snoring	230	335	0.935
	7.5 (3.0, 13.0)	8 (3.0, 13.0)	
Headache	248	337	< 0.001**
	5 (2.0, 11.0)	8 (4.0, 14.0)	
Frontal bossing	296	289	< 0.001**
	6 (3.0, 11.0)	9 (3.0, 15.0)	
Visual impairment	331	254	0.003**
	7 (2.0, 11.0)	8 (3.0, 15.0)	
Diastema	339	246	0.002**
	6 (2.0, 12.0)	9 (4.0, 14.0)	
Palpitations	413	172	< 0.001**
	6 (2.0, 12.0)	9 (4.6, 15.0)	
Malocclusion	444	141	0.042*
	7 (3.0, 15.0)	9 (3.0, 12.0)	
Visual field defects	449	136	< 0.001**
	7 (2.5, 12.0)	10 (4.0, 15.0)	
Arthralgia	364	221	0.114
	7 (3.0, 12.0)	8 (3.0, 15.0)	
Knee joint dysfunction	505	80	0.349
	8 (3.0, 13.0)	7 (2.0, 13.0)	
**Cognitive dysfunctions**			
Memory impairment	140	445	0.003**
	6 (2.0, 11.0)	8 (3.0, 14.0)	
Learning disability	458	127	< 0.001**
	6 (2.0, 12.0)	11 (7.0, 16.0)	
Dyscalculia	485	100	< 0.001**
	7 (3.0, 12.0)	11 (4.3, 16.0)	
Hallucinations	519	66	< 0.001**
	7 (3.0, 12.0)	12 (7.6, 17.3)	
Spatial perception disorder	532	53	0.006**
	7 (3.0, 12.0)	10 (6.0, 15.0)	
Illusions	533	52	< 0.001**
	7 (3.0, 12.0)	12 (9.0, 16.0)	
**Comorbidities**			
Menstrual irregularity	117	231	0.006**
	6 (2.0, 11.0)	8 (3.0, 14.0)	
Sexual dysfunction	361	224	0.005**
	5 (2.0, 11.0)	7 (5.0, 15.0)	
Obstructive sleep apnea	443	142	0.529
	7 (3.0, 13.0)	8 (3.0, 14.0)	
Obesity	485	100	0.009**
	7 (3.0, 12.0)	9 (4.3, 15.0)	
Colonic diseases	506	79	< 0.001**
	4 (3.0, 11.0)	9 (5.0, 16.0)	
Vertebral fracture	576	9	0.056
	7 (3.0, 13.0)	11 (9.0, 14.0)	

*Note:* *p < 0.05, **p < 0.01.

### 3.5 Low‐ Versus High‐IIP‐D Groups

Compared with their low‐score counterparts, patients in the high‐IIP‐D group (score ≥ 17) more frequently exhibited acral enlargement (*p* = 0.011), zygomatic hypertrophy (*p* = 0.010), headaches (*p* = 0.044), frontal bossing (*p* = 0.003), visual impairment (*p* = 0.004), palpitations (*p* = 0.002), and visual field defects (*p* = 0.037). They also had higher rates of memory impairment (*p* = 0.013), learning disability, dyscalculia, hallucinations (all *p* < 0.001), illusions (*p* = 0.047), menstrual irregularity (*p* = 0.036), sexual dysfunction (*p* = 0.027), and obesity (*p* = 0.002) (Table 2).

**TABLE 2:** Comparison of clinical characteristics and symptom burden between patients with high and low IIP‐D groups.

Variable	Condition	IIP‐D score		Total, *n* (%)	*P*
		High IIP‐D Group, *n* (%)	Low IIP‐D Group, *n* (%)		
Sex	Female	302 (59.0)	45 (61.6)	347 (59.3)	0.665
	Male	210 (41.0)	28 (38.4)	238 (40.7)	
Coarsening of facial features	Absence	34 (6.6)	3 (4.1)	37 (6.3)	0.406
	Presence	478 (93.4)	70 (95.9)	548 (97.7)	
Acral enlargement	Absence	42 (8.2)	0 (0)	42 (7.2)	0.011**
	Presence	470 (91.8)	73 (100)	543 (92.8)	
Zygomatic bone hypertrophy	Absence	201 (39.3)	18 (24.7)	219 (37.4)	0.010**
	Presence	311 (60.7)	55 (75.3)	366 (62.6)	
Snoring	Absence	203 (39.6)	27 (37.0)	230 (39.3)	0.663
	Presence	309 (60.4)	46 (63.0)	355 (60.7)	
Headache	Absence	225 (43.9)	23 (31.5)	248 (42.4)	0.044*
	Presence	287 (56.1)	50 (68.5)	337 (57.6)	
Frontal bossing	Absence	271 (52.9)	25 (34.2)	296 (50.6)	0.003**
	Presence	241 (47.1)	48 (65.8)	289 (49.4)	
Visual impairment	Absence	301 (58.8)	30 (41.1)	331 (56.6)	0.004**
	Presence	211 (41.2)	43 (58.9)	254 (43.4)	
Diastema	Absence	299 (58.4)	40 (54.8)	339 (57.9)	0.560
	Presence	213 (41.6)	33 (45.2)	246 (42.1)	
Palpitations	Absence	373 (72.9)	40 (54.8)	413 (70.6)	0.002**
	Presence	139 (27.1)	33 (45.2)	172 (29.4)	
Malocclusion	Absence	394 (77.0)	50 (68.5)	444 (75.9)	0.114
	Presence	118 (23.0)	23 (31.5)	141 (24.1)	
Visual field defects	Absence	400 (78.1)	49 (67.1)	449 (76.8)	0.037*
	Presence	112 (21.9)	24 (32.9)	136 (23.2)	
Arthralgia	Absence	323 (63.1)	41 (56.2)	364 (62.2)	0.254
	Presence	189 (36.9)	32 (43.8)	221 (37.8)	
Knee joint dysfunction	Absence	441 (86.3)	64 (87.7)	505 (86.3)	0.720
	Presence	71 (13.9)	9 (12.3)	80 (13.7)	
Memory impairment	Absence	131 (25.6)	9 (12.3)	140 (23.9)	0.013*
	Presence	381 (74.4)	64 (87.7)	445 (76.1)	
Learning disability	Absence	413 (80.7)	45 (61.6)	458 (78.3)	< 0.001**
	Presence	99 (78.0)	28 (22.0)	127 (21.7)	
Dyscalculia	Absence	435 (85.0)	50 (68.5)	485 (82.9)	< 0.001**
	Presence	77 (15.0)	23 (31.5)	100 (17.1)	
Hallucinations	Absence	465 (90.8)	54 (74.0)	519 (88.7)	< 0.001**
	Presence	47 (9.2)	19 (26.0)	66 (11.3)	
Spatial perception disorder	Absence	469 (91.6)	63 (86.3)	532 (90.9)	0.140
	Presence	43 (8.4)	10 (13.7)	53 (9.1)	
Illusions	Absence	471 (92.0)	62 (84.9)	533 (91.1)	0.047*
	Presence	41 (8.0)	11 (15.1)	52 (8.9)	
Menstrual irregularity	Absence	318 (62.1)	36 (49.3)	354 (60.5)	0.036*
	Presence	194 (37.9)	37 (50.7)	231 (39.5)	
Sexual dysfunction	Absence	262 (75.0)	99 (41.9)	361 (61.7)	0.027*
	Presence	87 (25.0)	137 (58.1)	224 (38.3)	
Obstructive sleep apnea	Absence	388 (75.8)	55 (75.3)	443 (75.7)	0.935
	Presence	124 (24.2)	18 (24.7)	142 (24.3)	
Obesity	Absence	434 (84.8)	51 (69.9)	485 (82.9)	0.002**
	Presence	78 (15.2)	22 (30.1)	100 (17.1)	
Colonic diseases	Absence	486 (91.2)	20 (38.5)	506 (86.5)	0.061
	Presence	47 (8.8)	32 (61.5)	79 (13.5)	
Vertebral fracture	Absence	503 (98.2)	73 (100)	576 (98.5)	0.254
	Presence	9 (1.8)	0 (0)	9 (1.5)	

*Note:* *p < 0.05, **p < 0.01.

### 3.6 Machine‐Learning‐Based Identification of Metabolic Factors Associated With Elevated IIP‐D Scores

#### 3.6.1 Feature Importance

**FIGURE 2:**
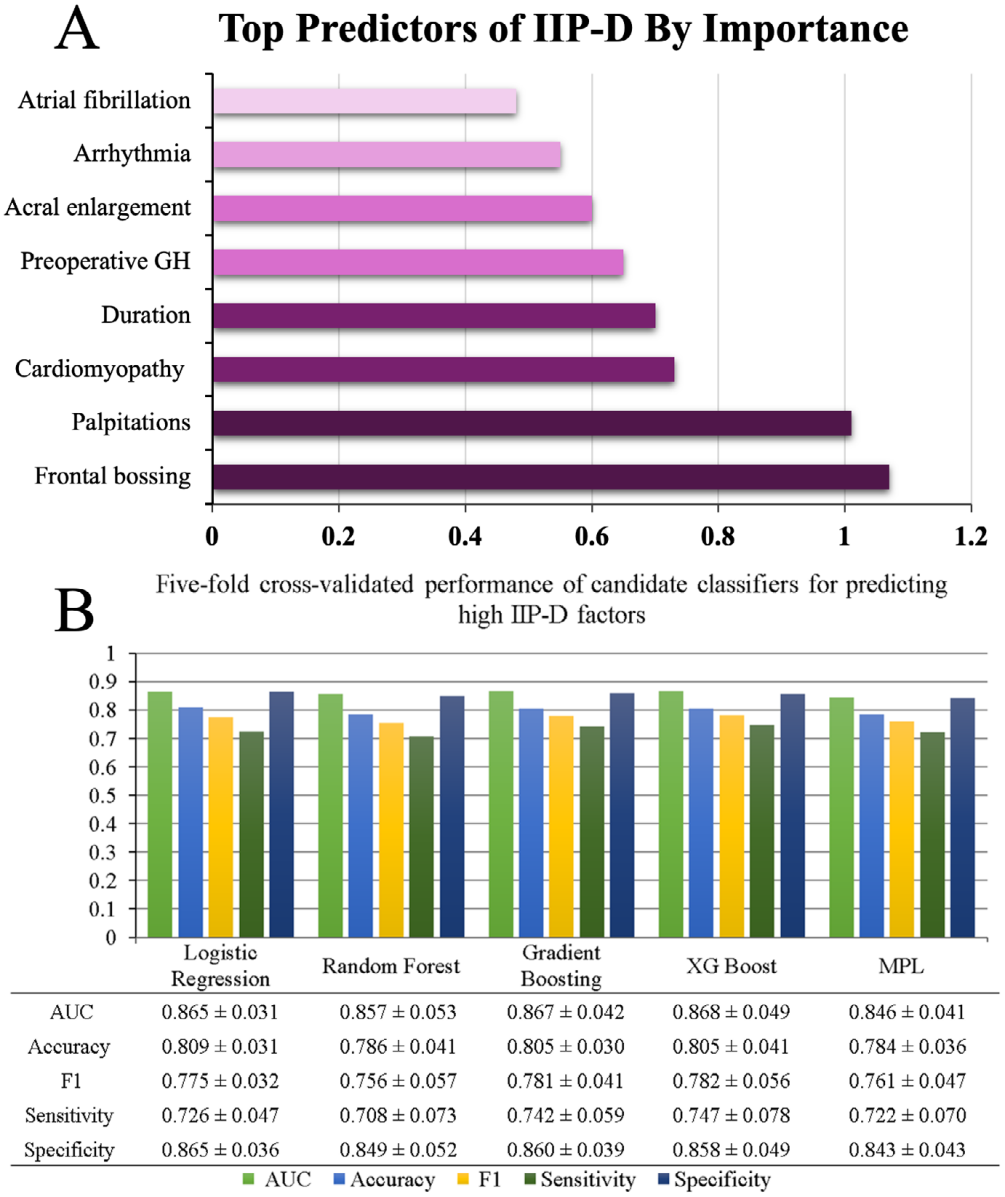
Machine‐learning model result visualization: (A) Top predictors of clinically significant interpersonal dysfunction (IIP‐D) in acromegaly patients, as determined by feature importance ranking from the multivariable machine‐learning model. The bar graph illustrates the relative importance of each predictor, with higher values indicating stronger associations with the high IIP‐D group. (B) Five‐fold cross‐validation performance of candidate classifiers for predicting the high IIP‐D factors: The performance of logistic regression, random forest, gradient boosting, extreme gradient boosting (XGBoost), and multilayer perceptron (MPL) models in predicting high interpersonal dysfunction (IIP‐D ≥ 17) is shown. Model performance is evaluated using four metrics: AUC (green), accuracy (blue), F1‐score (yellow), sensitivity (dark green), and specificity (dark blue). XGBoost achieved the highest AUC (0.868), followed by gradient boosting (AUC = 0.867). All models demonstrate high specificity and varying sensitivity, with XGBoost providing the best overall performance. Data is presented as mean values from five‐fold cross‐validation.

Based on the logistic regression model, the most influential predictors of high IIP‐D group were frontal bossing (10.7), palpitations (1.01), cardiomyopathy (0.73), disease duration (0.70), preoperative GH concentration (0.65), acral enlargement (0.60), arrhythmia (0.55), and atrial fibrillation (0.48) (Figure 2A).

#### 3.6.2 Prediction Model Performance

The candidate classifiers included logistic regression, random forest, gradient boosting, extreme gradient boosting (XGBoost), and multilayer perceptron (MLP). Across five‐fold cross‐validation, the highest AUCs were observed for gradient boosting (AUC = 0.867) and XGBoost (AUC = 0.868), with comparable high performance across metrics (Figure 2B). XGBoost was selected for final testing, achieving an AUC of 0.873, accuracy of 0.812, F1‐score of 0.789, sensitivity of 0.750, and specificity of 0.857.

### 3.7 Hierarchical Cluster Analysis

**FIGURE 3:**
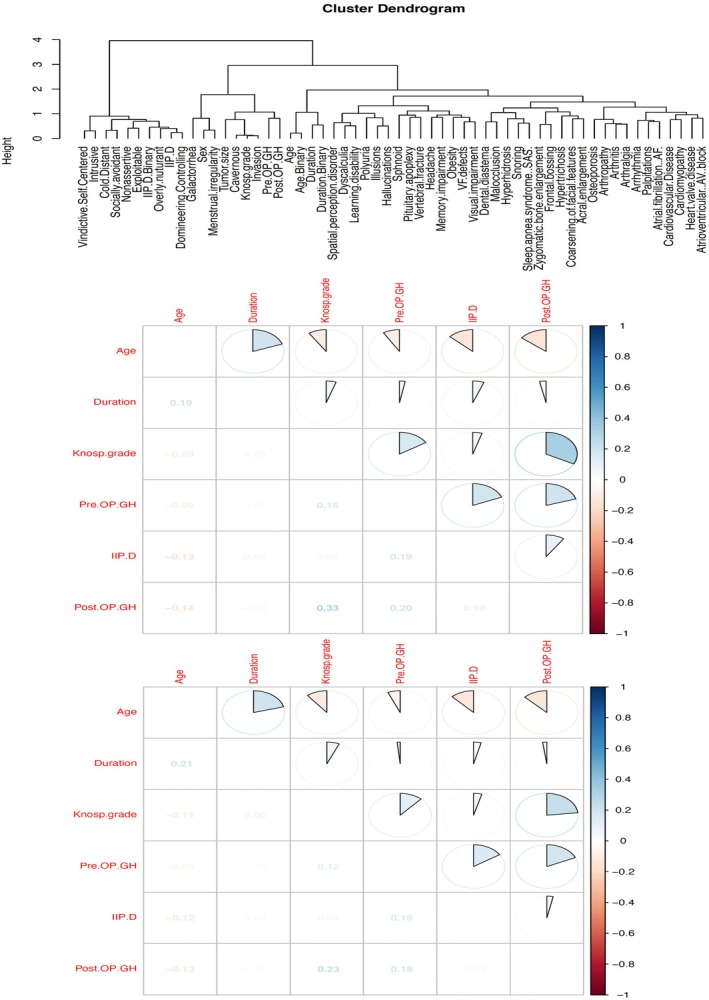
Spearman's and Pearson's correlations, evaluating the correlation between IIP‐D variables and clinical parameters.

Hierarchical cluster analysis (Figure 3) revealed three primary clusters, each representing distinct symptom profiles in patients with acromegaly.

#### 3.7.1 Cluster 1: Interpersonal and Behavioral Symptoms

Included interpersonal difficulties such as being vindictive/self‐centered, intrusive, socially avoidant, cold/distant, nonassertive, overly nurturant, exploitable, and domineering, reflecting a significant psychosocial burden.

#### 3.7.2 Cluster 2: Clinical Parameters and Cognitive Symptoms

The variables were grouped according to tumor characteristics (tumor size, Knosp grade, preoperative and postoperative GH levels, and cavernous sinus invasion), demographic characteristics (age and duration), cognitive dysfunction (spatial perception disorders, dyscalculia, and learning disabilities), menstrual irregularities, and galactorrhea. These features collectively suggest that clinical and cognitive complexities contribute to impaired social functioning.

#### 3.7.3 Cluster 3: Physical Manifestations and Cardiovascular Symptoms

The symptoms included pronounced physical changes (frontal bossing, zygomatic hypertrophy, and coarsening of facial features) and cardiovascular‐related symptoms (palpitations, atrial fibrillation, cardiovascular disease, AV block, and hypertension). This highlights the significant impact of somatic deformities and cardio‐cerebrovascular health on the patient's QoL and interpersonal relationships.

A moderate positive correlation was detected between Knosp grade and postoperative GH concentrations (Spearman |*ρ*| = 0.33; *p* < 0.001), indicating that cavernous‐sinus invasion is associated with higher residual GH secretion after transsphenoidal surgery (Figure 3). By contrast, all other pairwise correlations among age, symptom duration, tumor invasiveness, peri‐operative GH indices, and interpersonal dysfunction (IIP‐D) scores were weak (|*ρ*| < 0.25), underscoring the relative independence of these clinical and psychosocial domains.

## 4 Discussion

This nationwide cross‐sectional analysis of 585 Chinese acromegaly patients identified preoperative frontal bossing, palpitations, cardiomyopathy, duration, GH levels, and acral enlargement as robust, independent predictors of severe interpersonal dysfunction. These findings highlight the significant psychosocial burden in acromegaly, emphasizing that interpersonal difficulties are linked not only to visible physical changes but also to clinical symptoms reflecting systemic endocrine and cerebrovascular involvement.

Frontal bossing, indicative of pronounced skeletal disfigurement, also significantly predicted the interpersonal dysfunction factor in this study. This finding is particularly novel and significant because it highlights the psychological impact of visible physical abnormalities on social interaction. Such visible physical alterations can lead to stigma, reduced self‐esteem, and social anxiety, contributing directly to interpersonal avoidance [29, 30, 31, 32]. Additionally, frontal bossing serves as a marker of long‐standing GH exposure, indirectly representing cumulative cerebrovascular and cognitive damage.

Elevated GH levels emerged as another key predictor, consistent with prior research suggesting that chronic excess in GH levels is linked to structural and functional brain alterations, including reduced myelin‐sensitive signals and diffusion abnormalities across significant white matter tracts in patients with acromegaly [8]. Excess GH levels can downregulate endothelial nitric oxide bioavailability, promote oxidative stress, and diminish cerebral arteriole vasodilation [7]. These processes predispose patients to cerebral hypoperfusion, white matter lesions, and impaired neurovascular coupling, all contributing to cognitive deficits in memory, executive function, and emotional regulation [33, 34, 35, 36]. Consequently, patients with acromegaly may exhibit attenuated cerebrovascular reactivity and perfusion deficits, particularly in the watershed zones and white matter regions where collateral blood supply is tenuous. Furthermore, neurovascular coupling may be disrupted. Cerebrovascular disease (CVD) accounts for 2.4%–15.4% of all‐cause mortality in the patient population. CVD impacts the neurovascular changes, which negatively affect interpersonal functioning, providing a plausible mechanistic link between excess GH levels and psychosocial outcomes [4, 37].

CVD also triggers interactions mediated by the intrinsic cardiac nervous system (ICNS), making cardiac issues the second most prevalent and clinically significant problem in patients with acromegaly [4, 5, 38]. Autopsy studies have found that up to 93% of patients with acromegaly develop myocardial hypertrophy, and approximately 60% of patients experience cardiomyopathy, arrhythmia, hypertension, palpitations, or valvular heart disease during their lifetime [39]. The emergence of palpitations, cardiomyopathy, and atrial fibrillation (AF) as independent predictors highlights the role of cardiovascular dysfunction in acromegaly‐associated interpersonal difficulties, potentially mediated by psychological distress or anxiety associated with cardiac symptoms [40, 41]. Prior research indicates that in patients with acromegaly, cardiovascular symptoms can trigger anxiety and depressive disorders [42, 43]. Patients who are particularly sensitive to changes in heart rhythm often find themselves caught in a “chain reaction,” in which each episode of palpitations heightens their anxiety [21]. This increased anxiety can subsequently worsen the cardiac symptoms. The experience of recurrent palpitations frequently leads to heightened anxiety and hypervigilance, establishing a detrimental feedback loop between the heart and the central nervous system (CNS) [21, 42]. A study found that arrhythmias (such as AF and palpitations) illustrate this two‐way street: approximately one‐third of patients with AF exhibit elevated anxiety or depression, which in turn negatively affects their overall QoL [4, 6, 44]. Thus, there is a “heart–brain axis” at play; cardiac symptoms such as palpitations can aggravate CNS‐mediated conditions, while mental stress can exacerbate cardiac arrhythmias and palpitations [45].

Patients associated with palpitations for longer durations, mainly the patients with acromegaly, commonly reflect underlying arrhythmias or reduced cardiac function, leading to diminished cardiac output and cerebral hypoperfusion, which can exacerbate structural brain alterations, such as brain atrophy and white matter lesions [6, 46]. These alterations in the cortical regions are often associated with cognitive decline, particularly affecting memory, attention, and emotional stability. These findings underscore the cardiovascular–cerebrovascular interplay in acromegaly and suggest that cardiac evaluation and intervention could be critical components in managing psychosocial complications [47, 48, 49]. Our findings further indicate that patients with cardiac complications exhibited significantly higher IIP‐D scores, reflecting greater interpersonal dysfunction. This observation aligns with existing evidence suggesting that the burden of physical comorbidities exacerbates psychological distress and impairs social functioning in chronic endocrine disorders. For instance, a multicenter study on acromegaly revealed that both depression and arthropathy independently contributed to a diminished QoL [21, 33].

Clinically, these findings advocate the critical need for routine psychosocial evaluation within standard neuroendocrine care practices, particularly for patients with elevated preoperative GH levels, pronounced skeletal changes, and acral enlargement [1, 9, 15, 50, 51]. Incorporating systematic screening tools, such as the IIP‐D, into routine clinical assessments could significantly improve early identification and management strategies for patients at risk of interpersonal dysfunction, ultimately enhancing overall patient care and QoL.

However, the KMO value in our study is below the usual cutoff value of 0.438, commonly 0.5 for minimally acceptable adequacy [52], suggesting that our data set's inter‐item correlations may be too diffuse for a confident factor extraction. Despite the low KMO, we proceeded with the exploratory hierarchical cluster analysis because Bartlett's test of sphericity was highly significant (*χ* ^2^ = 18,324, *p* < 0.001), indicating that there were non‐random correlations among at least some variables in our cohort.

Our hierarchical cluster analysis further reinforced the multifaceted burden of acromegaly by delineating three primary symptom clusters. This stratification underscores the disease's multidimensional impact, reflecting how acromegaly affects psychosocial, neurocognitive, and systemic domains in parallel [15]. The first cluster, marked by pronounced interpersonal and behavioral difficulties, may benefit from early referral for psychological or psychiatric support to address issues like social withdrawal, depression, anxiety, and related interpersonal dysfunction [43, 53]. Targeted interventions aimed at reducing these psychosocial symptoms can significantly improve patients' well‐being [53]. By contrast, those in the second cluster (characterized by clinical parameters and cognitive impairments) would benefit from tailored neuropsychological evaluation, cognitive rehabilitation programs, and careful endocrine follow‐up to optimize hormonal control and cognitive function [15, 34].

Finally, the third cluster, dominated by severe physical manifestations and comorbidities (e.g., cardiovascular complications), requires vigilant management of cardiometabolic risk factors. Aggressive cardiovascular risk reduction and regular cardiac evaluations are crucial for patients [54, 55]. Recognizing which cluster an individual patient falls into can thus guide clinicians in formulating a comprehensive treatment plan emphasizing psychosocial support versus medical or cognitive interventions according to the patient's predominant needs [53]. This cluster‐driven perspective highlights that management of acromegaly should be highly individualized: different patients will require emphasis on various aspects of care (psychosocial vs. cognitive vs. cardiometabolic), beyond standard biochemical control, to effectively address the full spectrum of the disease's burden.

In this study, we evaluated the performance of several machine‐learning models to predict significant interpersonal dysfunction in high IIP‐D group patients. The candidate classifiers included logistic regression, random forest, gradient boosting, extreme gradient boosting (XGBoost), and multilayer perception (MLP). Across five‐fold cross‐validation, the highest AUCs were observed for gradient boosting. Among these models, XGBoost was selected for final testing due to its optimal performance. These results suggest that XGBoost outperformed other models in terms of overall classification accuracy and sensitivity, making it the most reliable model [56, 57]. The strongest predictors included frontal bossing, palpitations, cardiovascular complications, disease duration, preoperative GH levels, and acral enlargement.

However, several limitations should also be acknowledged. The retrospective cross‐sectional design precludes definitive conclusions regarding causality. Although broadly validated, the IIP‐D scale lacks specific validation in acromegaly populations, warranting caution when generalizing these findings. The suboptimal KMO may limit the reliability of the cluster analysis, and therefore any conclusions drawn from it (such as the existence of three distinct patient subgroups) must be considered provisional. The study's reliance on registry data introduces potential selection bias, and cultural factors specific to the Chinese population may limit its international generalizability. Future prospective studies incorporating neuroimaging and comprehensive neuropsychological evaluations are essential to clarify the underlying mechanisms and confirm these associations. Intervention trials assessing the efficacy of psychosocial treatment in patients with acromegaly are also highly beneficial.

## 5 Conclusions

This nationwide multicenter study demonstrates that preoperative GH levels elevation, cardiovascular symptoms (particularly palpitations), and frontal bossing were robust predictors of interpersonal difficulties in patients with acromegaly. These findings highlight that endocrine dysregulation, GH‐driven cerebrovascular changes, cardiovascular involvement, and visible skeletal deformities substantially impair social interactions and QoL. Integrating multidisciplinary routine psychosocial screening and timely interventions into standard clinical practice could markedly improve interpersonal functioning and overall patient outcomes. Future research should clarify the underlying neurobiological mechanisms and evaluate targeted therapeutic interventions. Timely interventions in standard clinical practice could markedly improve interpersonal functioning and overall patient outcomes. Future research should further elucidate the neurobiological mechanisms and evaluate the effectiveness of integrated therapeutic strategies.

## Author Contributions

S.Y.: conceptualization and design. S.K.T., Z.W., Y.L.L. and G.Z.: writing – original draft preparation, Y.T. and S.Y.: writing – reviewing and editing. S.K.T., Z.W., J.W., and S.Y.: formal analysis. W.S., Q.R., H.W., D.H., Y.M., Y.T., W.C., and S.Y.: results interpretation. S.K.T. and S.Y.: visualization. H.W., D.H., and S.Y.: supervision. W.C.: acquisition of data. All authors read and approved the final manuscript.

## Ethics Statement

This study was approved by the Institutional Review Board and Ethics Committee of The First Affiliated Hospital, Sun Yat‐sen University, and all patients provided electronic informed consent for the anonymous use of their data.

## Conflicts of Interest

The authors declare no conflicts of interest.

## Supporting information


**Table S1:** Baseline clinical, biochemical, and symptomatic characteristics of 585 patients with acromegaly.

## Data Availability

To uphold our commitment to patient privacy and address ethical considerations, we will provide only anonymized data that are not included in this article, available upon request to qualified investigators. Please contact the corresponding authors for data requests.
