# Supplementary material for: Metabolic Factors Related to Interpersonal Dysfunction in Acromegaly: A Nationwide Cross‐Sectional Study in China
Source: CNS Neurosci Ther. 2025 Sep 15;31(9):e70607. doi: 10.1111/cns.70607 (PMC12436684; doi:10.1111/cns.70607)
Supplement: Supplementary file 1 — Table S1: Baseline clinical, biochemical, and symptomatic characteristics of 585 patients with acromegaly. [file CNS-31-e70607-s001.docx]

**Supplementary Materials**

**Table 1: Baseline clinical, biochemical, and symptomatic characteristics of 585 patients with acromegaly.**

| Categories | Distribution |
| --- | --- |
| Mean age (years), mean ± SD | 37.6 ± 9.9 |
| Male | 238 |
| Female | 347 |
| Disease duration (years), median (IQR) | 4.7 (2.1 - 8.6) |
| Hormone profiles prior to surgery (mean ± SD) | |
| GH (μg/L) | 24.3 ± 19.8 μg/L |
| IGF-1 (μg/L) | 773 ± 295.1 μg/L |
| IGF-1 (ULN) | 3.5 ± 2.1 μg/L |
| Follow-up after initial diagnosis (months), median (IQR) | 53 (30 - 85) |
| Symptoms, n (%) | |
| Coarsening of facial features | 548 (93.7) |
| Acral enlargement | 543 (92.8) |
| Zygomatic bone hypertrophy | 366 (62.6) |
| Snoring | 355 (60.7) |
| Headache | 337 (57.6) |
| Hyperhidrosis | 326 (56.7) |
| Frontal bossing | 289 (49.4) |
| Visual impairment | 254 (43.4) |
| Diastema | 246 (42.0) |
| Arthralgia | 221 (37.8) |
| Palpitations | 172 (29.4) |
| Malocclusion | 141 (24.1) |
| Visual field defects | 136 (23.2) |
| Knee joint dysfunction | 80 (13.7) |
| Pituitary apoplexy | 56 (9.6） |
| Cognitive Dysfunctions, n (%) | |
| Memory impairment | 445 (76.1) |
| Learning disability | 127 (21.7) |
| Dyscalculia | 100 (17.1) |
| Spatial perception disorder | 53 (9.0) |
| Psychological Symptoms, n (%) | |
| Hallucinations | 66 (11.3) |
| Illusions | 52 (8.9) |
| Comorbidities, n (%) |  |
| Menstrual irregularity | 231 (66.3) |
| Sexual dysfunction | 224 (38.3) |
| Obstructive sleep apnea | 142 (24.3) |
| Obesity | 100 (17.1) |
| Colonic disease | 79 (13.5) |
| Vertebral fracture | 9 (1.5) |
| Remission status, n (%) | |
| Achieved biochemical remission | 224 (38.3) |
| Biochemically active | 361 (61.7) |
| Tumor size, n (%) | |
| Microadenoma (< 1cm) | 130 (22.2) |
| Macroadenoma (1 - 4cm) | 397 (67.7) |
| Giant adenoma (≥ 4cm) | 58 (9.9) |
| Knosp grade, n (%) | |
| 0-2 grade | 213 (36.4) |
| 3-4 grade | 372 (63.5) |
| Immunohistochemical subtype, n (%) | |
| GH | 476 (81.4) |
| GH + PRL | 78 (13.3) |
| GH + TSH | 5 (0.9) |
| GH + ACTH | 4 (0.5) |
| GH + (> 2 Immunohistochemical subtypes) | 22 (3.8) |

Values are presented as mean ± standard deviation (SD), median with interquartile range (IQR), or number and percentage (*n*, %). Symptom data include somatic, cognitive, and psychological domains based on clinical evaluation and patient-reported outcomes.
